# Expansion and Compression of Time Correlate with Information Processing in an Enumeration Task

**DOI:** 10.1371/journal.pone.0135794

**Published:** 2015-08-26

**Authors:** Andreas Wutz, Anuj Shukla, Raju S. Bapi, David Melcher

**Affiliations:** 1 Center for Mind & Brain Sciences, University of Trento, Rovereto, Italy; 2 Cognitive Science Laboratory, International Institute of Information Technology, Hyderabad, India; University of Cambridge, UNITED KINGDOM

## Abstract

Perception of temporal duration is subjective and is influenced by factors such as attention and context. For example, unexpected or emotional events are often experienced as if time subjectively expands, suggesting that the amount of information processed in a unit of time can be increased. Time dilation effects have been measured with an oddball paradigm in which an infrequent stimulus is perceived to last longer than standard stimuli in the rest of the sequence. Likewise, time compression for the oddball occurs when the duration of the standard items is relatively brief. Here, we investigated whether the amount of information processing changes when time is perceived as distorted. On each trial, an oddball stimulus of varying numerosity (1–14 items) and duration was presented along with standard items that were either short (70 ms) or long (1050 ms). Observers were instructed to count the number of dots within the oddball stimulus and to judge its relative duration with respect to the standards on that trial. Consistent with previous results, oddballs were reliably perceived as temporally distorted: expanded for longer standard stimuli blocks and compressed for shorter standards. The occurrence of these distortions of time perception correlated with perceptual processing; i.e. enumeration accuracy increased when time was perceived as expanded and decreased with temporal compression. These results suggest that subjective time distortions are not epiphenomenal, but reflect real changes in sensory processing. Such short-term plasticity in information processing rate could be evolutionarily advantageous in optimizing perception and action during critical moments.

## Introduction

One common observation about time perception is that it is subjective, dependent on factors such as attention, mood, and memory rather than only the objective passing of time measured by a clock (for review, see [1–3]). Although time is generally assumed to be a property of the external physical world, it cannot be sensed directly like light or sound. There is currently no consensus on the mechanisms that underlie judgments of perceived time, or how such mechanisms might underlie subjective illusions and errors in time tasks (for review, see [3]). Nonetheless, phenomenological experiences of time expanding or contracting are reported frequently and replicated experimentally.

One classic example of the subjectivity of our perception of elapsed time is that unexpected, dangerous or emotional events can be experienced to last longer than normal (for review, see [4]). While it is possible to measure these effects in naturalistic situations, such as skydiving [5] or bungee jumping [6], time dilation has more commonly been measured in the laboratory using an “oddball” paradigm [7–10]. In such experiments, an infrequent stimulus is perceived to last longer than the standard stimuli in the rest of the sequence. In contrast, time compression for the oddball occurs when the duration of the standards in the sequence is relatively brief [7].

According to classic models of temporal duration perception which posit the existence of an internal clock or pacemaker ([11–13], for a recent review see [14]), a novel or arousing event leads to the accumulation of more “ticks” in the internal clock or an increase in the rate of the pacemaker and thus longer perceived durations [8, 10]. However, the link between arousal and cognitive processing is complex and remains a matter of debate. Arousal may increase or decrease the perceived duration of events, depending on the time of arousal and the context [5]. In theory, it would be useful for time to effectively slow down during an emergency if this would actually allow us to think or do more in the same amount of external, clock time. This raises the question whether during perceived time expansion the brain is actually capable of doing more in the same amount of (objective) time.

Some evidence for more/better cognitive processing when time is subjectively expanded comes from a paradigm that used “click trains” to alter the perceived rate of the passage of time [15]. Previous studies have reported that the presence of a train of regular clicks increases the subjective duration of a subsequent stimulus [16]. Participants in that study made faster responses in reaction time tasks and reported more correct items in an iconic memory and a scene memory task [15]. These results are consistent with improved information processing when time is dilated. However, one limit of that study is that the authors only compared conditions with a preceding click train to those with silence and did not directly measure or quantify the presence of temporal expansion on any particular trial. The perceived expansion of time was inferred based on previous studies using click trains, but not directly tested. Thus, it is possible that some additional aspect of the presentation of the clicks, such as an increase in general arousal levels or temporal expectation, rather than temporal expansion, was related to the improvement in performance.

In order to further test the link between time perception and information processing in the context of time expansion and compression in an oddball paradigm, we measured the perceived duration of events while also tracking the accuracy on each trial. We used an enumeration task since performance on this task improves with longer presentation durations. Outside of the subitizing range of 1–4 items, observers typically make enumeration errors when they respond quickly [17, 18], supposedly because processing exceeds working memory capacity limits to the number of items that can be apprehended at once (for review see [19]). Counting requires processing over an extended temporal interval in order to perform successive perceptual steps, like indexing of salient items, marking previously indexed locations or shifting the processing focus [20]. Hence, processing of multiple items critically depends on the time to process input (for review, see [21]). If temporal expansion actually reflects the ability to perceive/think/do more, or do it more effectively, in the same amount of time, then we would expect a specific pattern of results in which participants are more likely to correctly enumerate items beyond the subitizing range when time is expanded and make more errors when they experience temporal compression.

The overall goal of our study was to test the claim that temporal expansion/compression is correlated, on a trial-by-trial basis, with better/worse performance in a visual cognition task. To this end, we use a prospective duration task in which participants report whether the oddball stimulus seems longer or shorter than the rest of the reference stimuli in the sequence (for reviews see [1] or [22]). Participants reported both the perceived duration and the number of dots in the oddball stimulus on each trial (the order of response was counterbalanced in experiments 1 and 2). In the first two experiments, we measured temporal expansion of stimulus durations around 1 s (experiment 1) and compression of durations around 100 ms (experiment 2) using a range of oddball durations centered around objective clock time [8,10]. Experiments 3 (long stimulus durations) and 4 (short stimulus durations) replicated the method of Tse and colleagues, which centered the oddballs around subjectively perceived duration [7]. In the fifth experiment, we presented oddballs at the point of subjective equality in order to directly compare enumeration accuracy in approximately equal numbers of trials with longer and shorter time judgments. Together, these five experiments allowed us to characterize the relationship between temporal distortions and performance on the enumeration task.

The exact mechanisms underlying the perception of duration, in particular whether it involves a dedicated and central internal clock/pacemaker [11–13] or rather a more distributed and non-specialized system that is used for making duration judgments [1–3], is beyond the scope of the present study (for review see [23]). Instead we aimed to further elucidate the nature of temporal distortions by directly testing the idea that perceived duration and information processing were correlated on a trial-by-trial basis.

## Materials and Methods

### 2.1. Subjects

Sixteen participants (10 female; mean age *M* = 23.6 years, *SD* = 2.2 years) took part in experiments 1 (long stimulus durations) and 2 (short stimulus durations). Two different groups of 15 (9 female; mean age *M* = 24.2 years, *SD* = 2.1 years) and 13 subjects (8 female; mean age *M* = 24.0 years, *SD* = 2.0 years) participated in experiments 3 (long stimulus durations) and 4 (short stimulus durations). Finally, 13 participants (9 female; mean age *M* = 23.3 years, *SD* = 1.8 years) were recruited to study the effects of temporal expansion around the point of subjective equality (PSE, experiment 5) between oddball and standard stimuli. All of the subjects provided written informed consent to the study. The ethics committee of the University of Trento approved this study. Subjects had normal or corrected-to-normal vision and received a small payment.

### 2.2. Stimuli and apparatus

**Fig 1:**
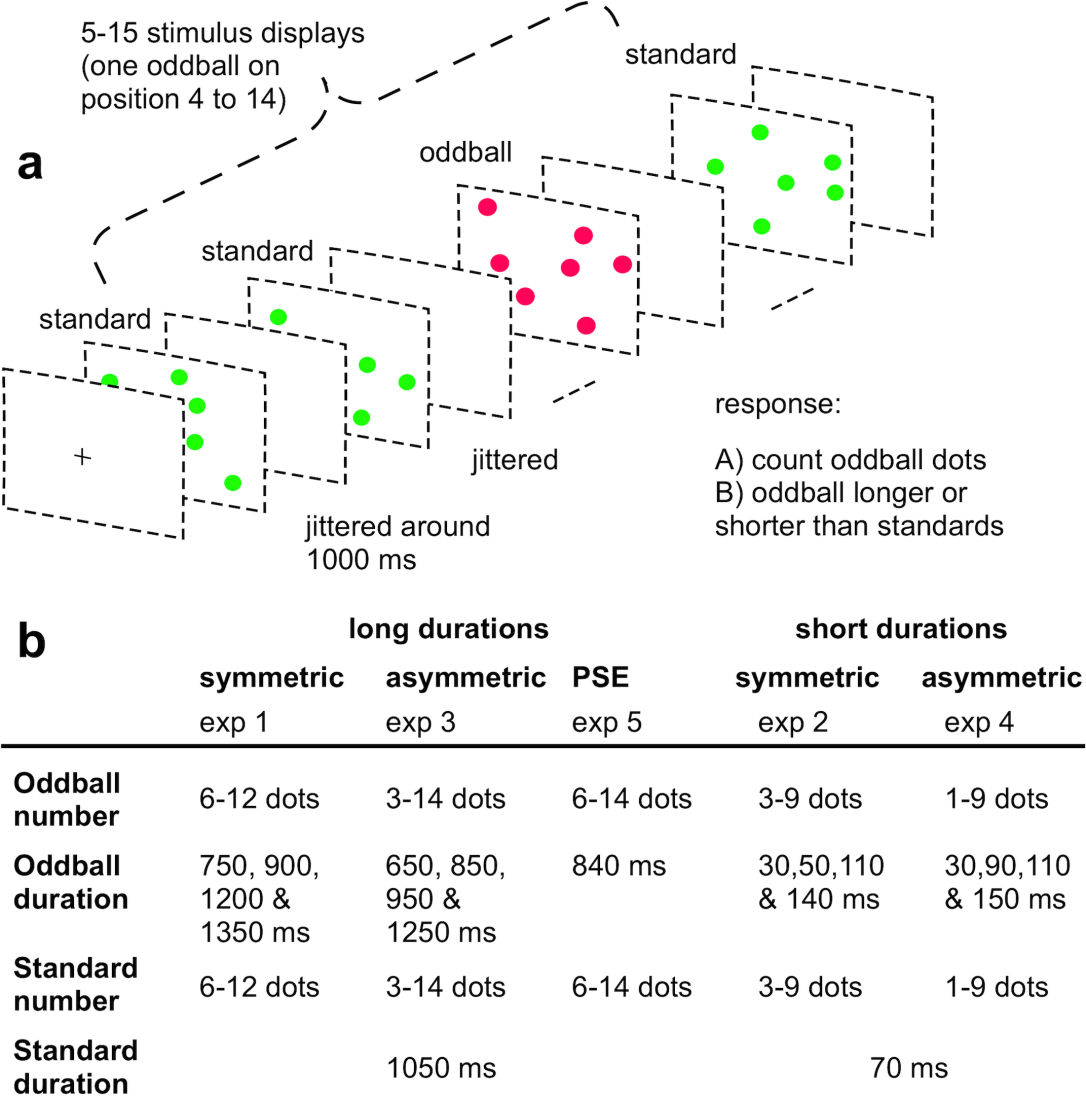
Illustration of the oddball procedure. a: Display sequence in one sample trial. On each trial a pseudo-randomized stream of 5 to 15 stimulus displays was shown, each interspersed with a randomly jittered blank inter-stimulus interval. A minimum of three standard displays containing green dots were presented before the first oddball occurrence made up of red dots and at least one standard followed an oddball on each trial. At the end of each trial sequence participants indicated the perceived number of red dots and judged its temporal duration relative to the standards. b: Experimental parameters for long stimulus durations (experiments 1 & 3), short stimulus durations (experiments 2 & 4) and long stimulus duration around the PSE (experiment 5). In all five experiments oddball number and duration were independently varied, in order to measure both enumeration and perceived duration on different temporal scales (around one second durations for temporal expansion and 100 ms durations for compression). Standard displays were always presented for a fixed duration and contained a pseudo-random number of items. In the fifth experiment temporal expansion was only examined for one oddball duration at the point of its subjective equality to standard duration.

The experiments were run on a Dell Intel Xeon processor using MATLAB 7.11.1 (MathWorks, Natick, MA) and the Psychophysics Toolbox, Version 3 [24, 25]. Participants were seated in a dimly lit room, at a distance of 57 cm from a 19” ViewSonic monitor (1024 × 768 resolution) running at 100 Hz. On each trial, a random number of 5 to 15 images, each containing a variable number of colored dots (1–14 items, depending on the particular experiment; see Fig 1B; 1° visual angle apiece), were presented on a gray background. Standard display images contained green dots, whereas red dots made up the oddball display (Fig 1A). Within a display each dot was placed randomly at one of 64 possible locations within an invisible, central 8 x 8 grid (16° x 16° visual angle in eccentricity). A random jitter (max. +/- 0.33° from grid cell center) was added to the dot locations on each trial. Each dot was separated on average by 1° of visual angle (min 0.33°; max 1.66°) to prevent drawing of overlapping dots. The presentation of each dot display was separated by an inter-stimulus interval (ISI) showing a uniform gray screen.

### 2.3. Procedure

The same oddball paradigm was used in all five experiments, differing only in the specific experimental parameters (Fig 1). All subjects received verbal and written instructions about each task and completed one practice block at the beginning of each session. In all five experiments, each trial began with a central fixation cross (black, 1°) on a gray background until the participant pressed an arbitrary key. After this self-paced trial start the fixation cross remained on the screen for another 500 ms, before a random number of display frames ranging from 5 to 15 were presented, each separated by an ISI (randomly jittered in steps of 10 ms between 950 ms and 1150 ms duration). The position of the oddball within the display sequence was pseudo-randomized on each trial. The earliest occurrence of an oddball display could be on fourth position and at least one standard display always followed the presentation of the oddball (at latest on the 14^th^ position within a sequence of 15 stimuli). Standard displays always contained a random number of green dots (within different ranges depending on the particular experiment) and were presented for a fixed standard duration (Fig 1B). Four different oddball durations were used in experiments 1–4, each within the corresponding range of temporal expansion (around 1 s–stimulus durations; Expts 1 & 3) and compression (around 100 ms–stimulus durations; Expts 2 & 4), respectively.

In experiments 1 and 2 two oddball durations were longer and two shorter than the standard duration. They were placed relatively symmetrically around the standard duration, in a way to both sample the time course effectively and also avoid the appearance of an oddball effect if participants just guessed [10]. In experiment 1 oddball durations were centered +/- 150 and 300 ms around the standard duration (1050 ms). In experiment 2 oddball durations were equally spaced between half and double the standard (70 ms) and thus categorically symmetric but not on a metric scale. This arrangement was chosen to sample a wide enough range of durations, in order to draw accurate psychometric curves of perceived oddball duration as a function of oddball time, even on this faster time scale with oddball durations in the range of only tens of milliseconds. Experiments 3 and 4 focused on oddball durations below (for long stimulus durations) and above the standard duration (for short stimulus durations) in order to yield a comparable proportion of shorter/longer responses. This design intended to yield a symmetric arrangement of durations in subjectively perceived time [7]. In experiment 5 there was only one oddball duration, corresponding to the point of subjective equality (PSE) between standard and oddball durations estimated from experiment 3 (Fig 1B).

Additionally, in experiments 3–5 we stratified the range of oddball set sizes into four (or three, in Experiments 4 and 5) balanced and equally spaced ‘number bins’ (Expt 3: 3–5, 6–8, 9–11 and 12–14 items; Expt 4: 1–3, 4–6 and 7–9; Expt 5: 6–8, 9–11 and 12–14 items). The number of red oddball dots was drawn randomly from each bin. Stratified number bin and oddball duration were independently varied factors in experiments 3–5, which allowed us to control for the effect of oddball number. In experiments 1 and 2 oddball number was drawn randomly on each trial with on average more items in experiment 1 with long durations (6–12 dots) than in experiment 2 with short durations (3–9 dots) to compensate for different oddball presentation durations and match enumeration task difficulty across experiments.

Upon oddball occurrence, subjects performed two tasks: enumeration of the red oddball dots and judgment of the perceived oddball duration. Subjects were instructed to compare the oddball duration relative to the standards immediately preceding and following the oddball. In experiments 1 and 2, both number and time judgments had to be spoken aloud after oddball occurrence and indicated manually at the end of the trial by pressing a number key and a key corresponding to oddball duration being shorter or longer than the standard duration. The use of vocal responses in studies of enumeration aims to force participants to respond quickly rather than slowly count or use other strategies. Subjects were instructed to report the same vocal and manual time and number judgment based on their “perceived” quantity rather than information recalled after memory-related processes at the end of the trial. Vocal and manual responses corresponded almost perfectly for both time and number judgments (on average 98.5% (+/-1.8% *SD*) across subject). Analysis was based on the vocal responses after correcting these few discrepancies. The response order (time or number judgment first) was alternated between blocks and counter-balanced across participants. In experiments 3–5 number judgments always preceded time judgments in order to measure reaction times for perceived oddball number with a voice key response (Sennheiser e835S). In addition, on each trial the subjects’ perceived number of dots was listed by the experimenter and analyzed for accurateness offline after the experiments. Participants reported their time judgment manually at the end of the trial.

Experiments 1 and 2 were run in a single session (ca. 1.5 h duration) containing 8 blocks with 32 trials each (4 blocks each for long and short stimulus durations, respectively; counter-balanced order across participants). Each oddball duration was shown 8 times per block in random order. One session in experiment 3 with long stimulus durations comprised 10 blocks of 32 trials (8 trials per oddball duration in random order) and lasted approximately 2.5 hours. One session in experiment 4 with short stimulus durations comprised 10 blocks of 24 trials (6 trials per oddball duration in random order) and lasted approximately 1.5 hours. Experiment 5 on temporal expansion around the PSE consisted of 5 blocks of 30 trials and lasted around 45 minutes.

### 2.4. Data analysis

Two main measures were derived from this oddball paradigm on each individual trial: the accuracy in enumerating the number of red oddball dots and the perceived oddball duration compared to standard duration (Expt 1 & 3: 1050 ms; Expt 2 & 4: 70 ms). The paradigm was designed to examine the assumption of stochastic independence between these two binary outcome variables (correct/incorrect number, shorter/longer duration) on each single experimental trial. We hypothesized a systematic violation of this assumption reflected in significantly more probable co-occurrences of “correct and longer” as well as “incorrect and shorter” trials in our data sets.

## Results

### 3.1. Task performance in time and number judgments as a function of set size

Before analyzing these joint-probability distributions, we monitored whether task performance in enumeration and time judgments reflected the expected pattern: set-size specific decrease in counting accuracy in the enumeration task and increase in duration judgments as a function of physical oddball duration. Error bars in all Results figures indicate one standard error of the mean (*SEM*) for within-subject designs (similar to the description in [26]).

#### 3.1.1. Enumeration accuracy as a function of presented number

**Fig 2:**
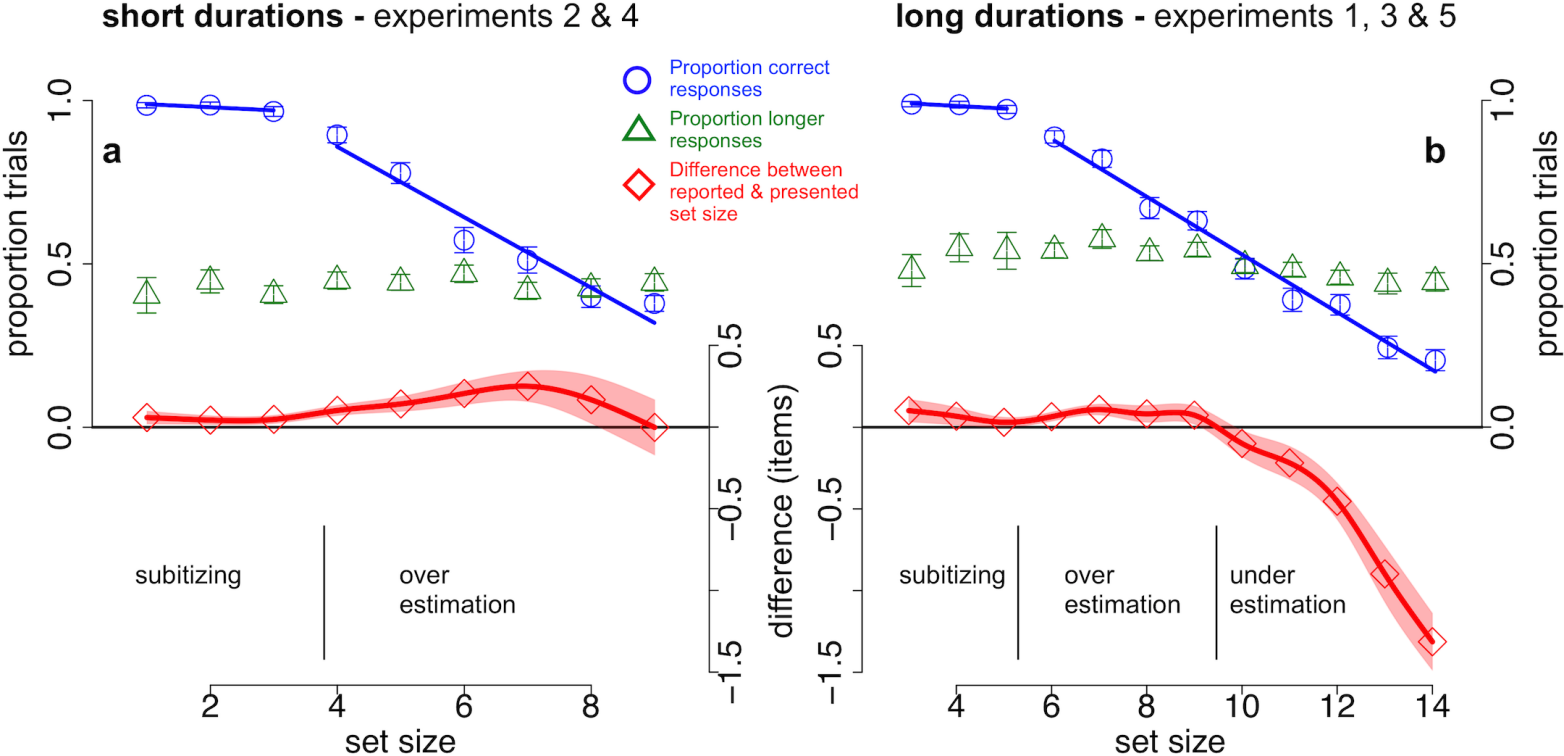
Time and number task performance as a function of set size. The proportion of correct responses (blue circles), proportion of longer responses (green triangles) and difference between reported and presented number (red rectangles) across different set sizes for short stimulus durations (a) and long stimulus durations (b). The proportion of correct responses remained close to ceiling for low set sizes and decreased linearly with every additional item thereafter. Similarly, this subitizing pattern was reflected in negligible differences between reported and presented low numbers (lower axis insets). Enumeration errors predominantly reflect overestimation of intermediate and underestimation of high set sizes (red). The proportion of longer responses (green) remained relatively stable across set sizes. Solid, straight lines show linear fits on the data (blue). Error bars and shaded regions indicate one standard error of the mean (*SEM*).

Across all five experiments, enumeration accuracy was around the 50% threshold, yielding an about equal proportion of correct and incorrect responses across different oddball durations and dot numbers (Expt. 1: 56.9%, *SD* = 13.5%; Expt. 2: 62.4%, *SD* = 13.8%; Expt. 3: 62.5%, *SD* = 15.1%; Expt. 4: 60.1%, *SD* = 9.6% (accuracy excluding small set sizes for Expt. 3 and 4); Expt. 5: 43.9%, *SD* = 9.7%). In experiments 3 and 4 (with an asymmetric design), in which we measured accuracy across stratified bins of dot numbers, we observed a ceiling effect for small numbers of items. In both experiments, accuracy and reaction times (RT) showed a clear “subitizing” pattern [17, 18] for low dot numbers (Expt. 3: 3–5 items; Expt. 4: 1–3 items): minimal differences in accuracy (P_cor_) and RT in correct trials within small set sizes but a significant drop in both measures between low and high numbers (Expt. 3: P_cor,low_ = 98.2%, *SD* = 2.9%; P_cor,high_ = 62.5%, *SD* = 15.1%; t-test P_cor,low_ vs. P_cor,high_: *t*(14) = 10.3, *p* < .001; RT_cor,low_ = 1.07 s, *SD* = .21 s; RT_cor,high_ = 2.1 s, *SD* = .43 s; *t*(14) = -13.3, p < .001; Expt. 4: P_cor,low_ = 98.1%, *SD* = 2.3%; P_cor,high_ = 60.1%, *SD* = 9.6%; *t*(12) = 13.4, p < .001; RT_cor,low_ = .75 s s, *SD* = .13 s; RT_cor,high_ = 1.3 s, *SD* = .26 s; *t*(12) = -9.1, p < .001; all reported *p*-values Bonferroni-corrected for multiple comparisons). Because of this ceiling effect we excluded these low set sizes from subsequent analyses for experiments 3 and 4. For higher set sizes, enumeration accuracy decreased linearly for 8.8% for longer (Expt. 1,3 & 5) and 10.8% for shorter oddball durations (Expt. 2 & 4) with every additional item to be counted (Expt. 1,3 & 5: linear fit adjusted R^2^ = .98; Expt. 2 & 4: linear fit adjusted R^2^ = .93; Fig 2).

#### 3.1.2. Reported number as a function of presented number

When observers did make enumeration errors, they tended to overestimate intermediate set sizes especially with shorter stimulus durations and underestimate high set sizes with longer stimulus durations (Fig 2). For longer stimulus durations (experiments 1,3 & 5), the difference between reported and presented number judgments (D) was not different from zero for low set sizes (3–5 items; t-test D vs 0, *t*(14) = 1.8, *p* < n.s.) but significantly positive for the intermediate set sizes 6 to 9 (*t*(43) = 2.7, *p* < .03) and significantly negative for high set sizes 10 to 14 (*t*(43) = -4.5, *p* < .001). Similarly for shorter stimulus durations (Expt. 2 & 4) response differences for low set sizes (1–3 items) did not differ from zero (*t*(28) = 1.8, *p* < n.s.) but were significantly positive for intermediate set sizes 4 to 8 (*t*(28) = 2.7, *p* < .03; all reported *p*-values Bonferroni-corrected for multiple comparisons). This pattern of enumeration data suggests that observers were generally able to “subitize” low set sizes, overestimated intermediate numerosities and failed to converge towards the correct number when serially counting high numbers of items for long presentation durations.

#### 3.1.3. Time judgments as a function of presented and perceived number

The proportion of longer responses remained relatively stable across set sizes at on average 50.8% (*SD* = 16.1%) for longer stimulus durations (Expt. 1,3 & 5) and 43.5% (*SD* = 14.4%) for shorter stimulus durations (Expt. 2 & 4; Fig 2). For longer stimulus durations, however, there was a consistent trend to perceive intermediate set sizes (6 to 9 items) more often as longer than the standard duration compared to high (10 to 14) numbers of items (one-way ANOVA, main effect of set size: *F*(11, 354) = 3.4, p < .001; intermediate vs. high *t*(43) = 3.5, *p* < .001). However, time judgments in low set sizes (3–5 items) did not statistically differ compared to neither intermediate (*t*(14) = -1.3, *p* < n.s.) nor high sets sizes (*t*(14) = .5, *p* < n.s.). For shorter stimulus durations there was no effect of set size on time judgments (*F*(8, 192) = .8, p < n.s.). In addition, there was a robust effect on the proportion of longer responses as a function of reported number for the longer stimulus durations (one-way ANOVA with levels underestimation (D<0), correct enumeration (D = 0) and overestimation (D>0): *F*(2,84) = 11.2, *p* < .001). Longer time judgments, however, were not associated with either error pattern (under- or overestimation: D<0 vs. D>0 *t*(43) = -.3, *p* = n.s.). Instead, participants judged oddballs more often as longer than the standard duration within correct enumeration trials (D<0 vs. D = 0 *t*(43) = -4.7, *p* = .001; D>0 vs. D = 0 *t*(43) = -3.4, *p* = .004; all reported *p*-values Bonferroni-corrected). For shorter stimulus durations we did not observe an effect of reported number on time judgments (*F*(2, 56) = 1.8, p < n.s.). In sum, time judgments did not depend linearly on the number of items on the screen—i.e. neither monotonically increasing nor decreasing time judgments with more or less items, respectively—or on the enumeration task difficulty. Likewise, perceived duration did not depend on numerical under- or overestimation. Thus, there was not a direct mapping between temporal and numerical magnitude judgments, although there were indications of a fairly small and complex interaction between these two factors.

### 3.2. Time judgments & enumeration accuracy as a function of oddball time

#### 3.2.1. Psychometric thresholds with symmetrically placed oddballs

**Fig 3:**
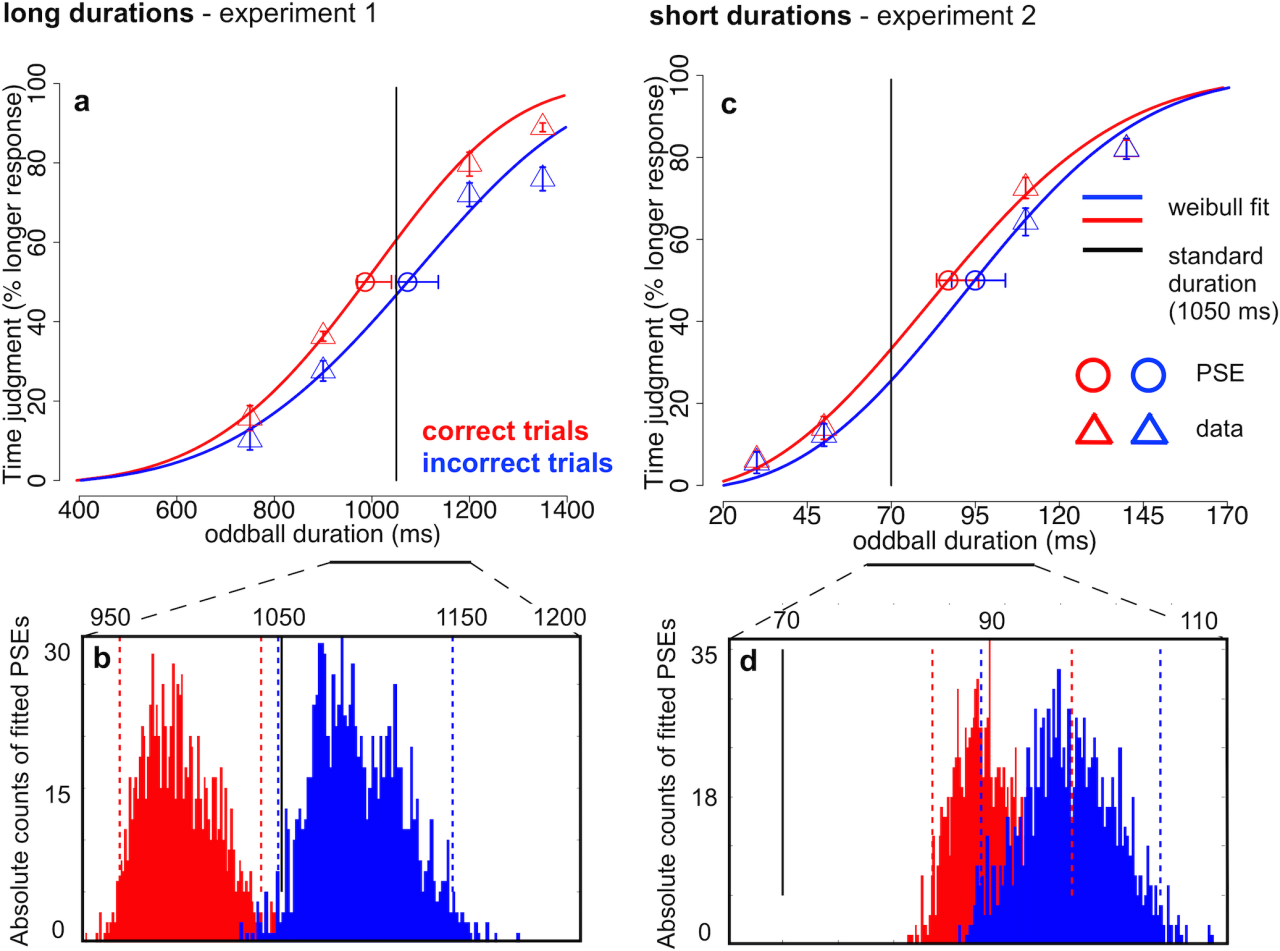
Psychometric curves of time judgments across oddball durations for experiments 1 & 2. a: Percent “longer” responses across stimulus durations around 1 s with a symmetric oddball placement (experiment 1) around the standard duration (1050 ms, black vertical line). The probability of responding “oddball > standard” increased with longer oddball durations for both correct (red triangles) and incorrect trials (blue triangles). The point of subjective equality (PSE, circles; 50% threshold of a Weibull fitted curve, solid lines) was only for correct trials significantly shorter than the standard duration. b: Absolute counts of PSEs resulting from 1000 bootstrap simulations for correct (red) and incorrect trials (blue). Only threshold estimates from correct trials were significantly shorter than the standard duration (black vertical line) and also significantly shorter than PSEs from incorrect trials. c: Percent “longer” responses across stimulus durations around 100 ms with a symmetric oddball placement (experiment 2) around the standard duration (70 ms, black vertical line). The probability of responding “oddball > standard” increased with longer oddball durations (triangles) and the point of subjective equality (PSE, circles; 50% threshold of a Weibull fitted curve, solid lines) exceeded the standard duration significantly for both correct (red) and incorrect trials (blue). d: Absolute counts of PSEs resulting from 1000 bootstrap simulations for correct (red) and incorrect trials (blue). Threshold estimates from both correct and incorrect trials significantly exceeded the standard duration (black vertical line). (a & c) Error bars for real data (triangles) indicate one standard error of the mean (*SEM*) for within-subject designs (similar to the description in [26]). Individual proportions of longer responses of each subject have been centered on their average proportions of longer responses across conditions before calculating the *SE*. Error bars for estimated thresholds (circles) indicate 95% confidence intervals of the bootstrapped threshold distributions. (b & d) Dashed vertical lines indicate 95% confidence intervals of the bootstrapped threshold distributions [28].

Time judgments were highly accurate in experiments 1 (long stimulus durations) & 2 (short stimulus durations), which used a symmetric arrangement of oddball durations around the standard duration. The percentage of longer responses ranged between 15.0% and 86.5% (*SDs* = 10.1% & 9.7% in Expt. 1) and between 5.3% and 83.8% (*SD* = 7.6% & 9.8% in Expt. 2) from the shortest to the longest oddball duration (750 to 1350 ms in Expt. 1; 30 to 140 ms in Expt. 2; Fig 3). Enumeration performance across oddball durations remained between 56%-59% (*SD*s between 13.8% and 17.1%) in experiment 1 for long durations and between 58.2%-66.2% (*SD*s between 14.6% and 16.7%) in experiment 2 for short durations. In order to test for perceived temporal expansion or compression of oddballs relative to standard durations, as well as whether these distortions of perceived time were related to enumeration accuracy, we calculated the point of subjective equality (PSE) between oddball and standard durations. The PSE was defined as the point on the psychometric curve (fitted with a Weibull function) in which a given oddball duration was perceived longer than the standard on half of the trials (Fig 3). Psychometric curve fitting was performed separately for correct and incorrect trials (using the MATLAB based toolbox psignifit, version 2.5.6 which implements the maximum-likelihood method described by [27, 28]). Confidence intervals for the threshold estimates were found by the percentile bootstrap method based on 1000 simulations (see [28]). Weibull functions fitted the data sufficiently well (average *deviance (d)* between bootstrapped fits and real values between *d* = 1.1 and *d* = 1.6; < χ^2^_p < .95, df = 4_ = 9.5).

The PSEs were 986 ms (CI = [970 1040]) in correct trials and 1073 ms (CI = [1049 1136]) in incorrect trials for long oddball durations around 1 s, for which we expected to find temporal expansion (Fig 3A and 3B). For correct trials 99.7% of the 1000 bootstrapped PSEs were below the standard duration (= 1050 ms) but in incorrect trials only 2.8% of the PSEs were lower than standard. PSEs from correct trials were lower than incorrect trials in 99.8% of the bootstrapped simulations and repeating this sign-test on 1000 random permutations of the simulated thresholds yielded a highly significant result (*p* < .001). In sum, temporal expansion (reflected in lower PSEs) was only found in correct enumeration trials.

For short stimulus durations around 100 ms, we expected perceived temporal compression as shown by higher PSEs compared to standard duration. The PSEs were 87 ms (CI = [83 96]) in correct trials and 95 ms (CI = [88 104]) in incorrect trials (Fig 3C and 3D). For both correct and incorrect trials all PSE estimates in the bootstrap distributions exceeded the standard duration of 70 ms. Between conditions, more than 90% of the PSEs from incorrect trials exceeded the estimated thresholds from correct trials. Thus, we found strong temporal compression for both correct and incorrect trials with a marginally significant trend towards stronger compression in incorrect trials (*p* = .096, sign-permutation test).

#### 3.2.2. Psychometric thresholds with asymmetrically placed oddballs

Similar analyses were performed with data from experiment 3 (long stimulus durations) and 4 (short stimulus durations), in which we sampled a wider range of oddball durations below the standard duration for longer stimuli and above the standard duration for shorter stimuli, following the approach of Tse and colleagues [7]. In agreement with previous reports [10], we expected a generic inflation of time expansion and compression due to this asymmetric design. However, we also expected to be more sensitive to the correlation between time expansion & compression and enumeration accuracy. The asymmetric oddball placement should create more symmetry in reports of subjective time and yield more balanced numbers of shorter and longer judgments over trials.

**Fig 4:**
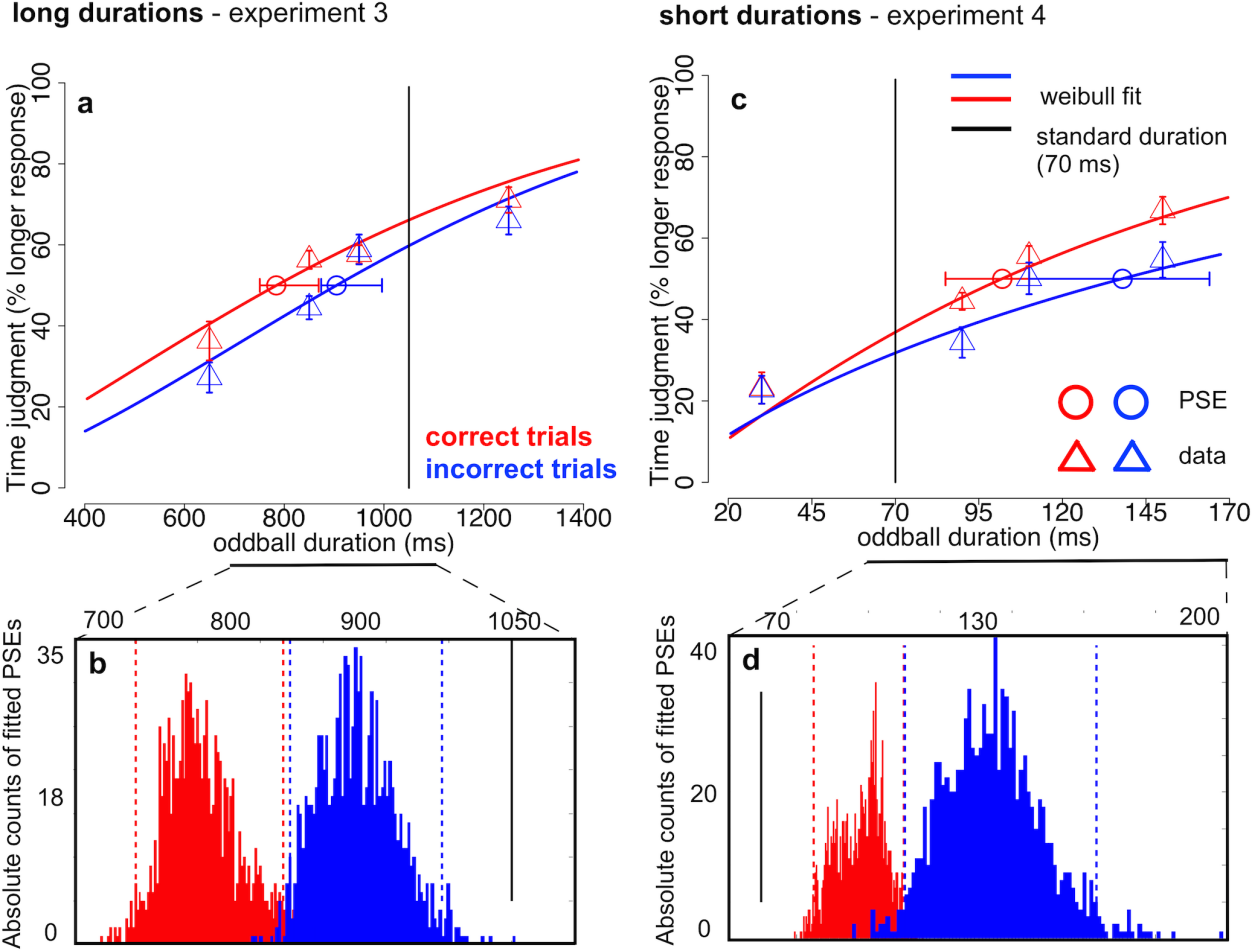
Psychometric curves of time judgments across oddball durations for experiments 3 & 4. a: Percent “longer” responses across stimulus durations around 1 s with an asymmetric oddball placement (experiment 3) around the standard duration (1050 ms, black vertical line). The probability of responding “oddball > standard” increased with longer oddball durations (triangles) and the point of subjective equality (PSE, circles; 50% threshold of a Weibull fitted curve, solid lines) was for both correct (red) and incorrect trials (blue) significantly shorter than the standard duration. b: Absolute counts of PSEs resulting from 1000 bootstrap simulations for correct (red) and incorrect trials (blue). Threshold estimates from both correct and incorrect trials were both significantly shorter than the standard duration (black vertical line). In addition, PSEs from correct trials were significantly lower than from incorrect trials. c: Percent “longer” responses across stimulus durations around 100 ms with an asymmetric oddball placement (experiment 4) around the standard duration (70 ms, black vertical line). The probability of responding “oddball > standard” increased with longer oddball durations (triangles) and the point of subjective equality (PSE, circles; 50% threshold of a Weibull fitted curve, solid lines) exceeded the standard duration significantly for both correct (red) and incorrect trials (blue). d: Absolute counts of PSEs resulting from 1000 bootstrap simulations for correct (red) and incorrect trials (blue). Threshold estimates from both correct and incorrect trials significantly exceeded the standard duration (black vertical line). PSEs from correct trials were significantly lower than from incorrect trials. (a & c) Error bars for real data (triangles) indicate one standard error of the mean (*SEM*) for within-subject designs (similar to the description in [26]). Individual proportions of longer responses of each subject have been centered on their average proportions of longer responses across conditions before calculating the *SE*. Error bars for estimated thresholds (circles) indicate 95% confidence intervals of the bootstrapped threshold distributions. (b & d) Dashed vertical lines indicate 95% confidence intervals of the bootstrapped threshold distributions [28].

In general, time judgments were less accurate. In experiment 3, observers judged oddballs that were considerably shorter than the standard duration (1050 ms) as longer on more than half of the trials (oddball duration 850 ms: 52.4%, *SD* = 10.9%; 950 ms: 56.2%, *SD* = 13.4%). In experiment 4, oddballs that were objectively longer than the standard (70 ms) were perceived as longer on about half of the trials (oddball duration 90 ms: 43.2%, *SD* = 10.5%; 110 ms: 52.4%, *SD* = 16.0%, Fig 4). Enumeration performance across oddball durations remained between 53%-65% (*SD*s between 14.9% and 18.1%) in experiment 3 for longer stimulus durations and between 57.1%-62.7% (*SD*s between 10.1% and 11.7%) in experiment 4 for shorter stimulus durations. Again, PSEs were derived from 50% thresholds of Weibull fits to the proportion of longer responses across oddball durations (average *deviance (d)* between bootstrapped fits and real values *d*<2; < χ^2^_p < .95, df = 4_ = 9.5).

For long stimulus durations around 1 s (Expt. 3) the PSEs were considerably lower than in experiment 1 which used a symmetric design. For correct trials we observed a PSE of 784 ms (CI = [751 869]) and for incorrect trials the PSE was 905 ms (CI = [874 996]; Fig 4A and 4B) and almost all simulated PSE estimates were below the standard duration (= 1050 ms) for both conditions (100% and 99.9% for correct and incorrect, respectively). As expected, temporal expansion was more prominent with an asymmetric than with a symmetric design but this bias resulted in a uniform leftward shift towards more expansion for both correct and incorrect response distributions. Between conditions stronger time expansion was again found in correct compared to incorrect trials (PSE_cor_ < PSE_incor_ = 99.9%, *p* < .002, permutation sign-test).

A similar pattern of stronger time compression with an asymmetric design was found for the short stimulus durations around 100 ms. The PSEs were 98 ms (CI = [85 109]) in correct trials and 134 ms (CI = [110 164]) in incorrect trials (Fig 4C and 4D). For both correct and incorrect trials all PSE estimate in the bootstrap distributions exceeded the standard duration of 70 ms. In contrast to the previous experiment 2, however, more than 99% of the PSEs from incorrect trials exceeded the estimated thresholds from correct trials signaling significantly stronger time compression when observers enumerated incorrectly (*p* < .005, permutation sign-test). In sum, we replicated the finding of perceived time compression for both correct and incorrect trials in experiment 2 but with an even stronger temporal compression effect in incorrect trials.

#### 3.2.3. Logistic and linear models of accuracy on time judgment

In order to pin these effects down statistically, we modeled the likelihood of a longer/shorter response on each individual trial within a mixed model logistic regression (Generalized Linear Mixed Models for binomially distributed outcomes, [29–31]) in all data sets. Logistic models were calculated using the statistics software R, Version 3.0.2 (R core team, 2013) and in particular the libraries *‘lme4’* [32] and *‘rms’* [33]. First, we formulated a “full model” containing a subject-specific intercept (S) and slope across oddball durations (T|S) as random effects and all main effects and interactions between the fixed factors enumeration accuracy (Acc: correct, incorrect) and physical oddball duration (durations around 1 s for experiments 1 & 3; durations around 100 ms for experiments 2 & 4). In experiments 1 and 2 we additionally controlled for the response order of the tasks (order: time or number judgment first). For experiments 3–5, in which we measured accuracy across stratified bins of dot numbers, we also included the factor set size in the full model formulas. Subsequently, we reduced the model formula to a sufficiently fitted model by identifying redundant model predictors using likelihood ratio tests (LRT). We successively removed the third- and second-order interactions and main factors—in case they did not contribute significantly to the model prediction (as assessed by Wald-z tests)—and tested each new model’s data log-likelihood against the full model. The −2 * logarithm of the ratio between the likelihoods of two models converges asymptotically to a *Χ* ^*2*^—distribution with its degrees of freedom (*df*) given by the difference between the number of parameters of each model [30]. LRTs therefore take the number of free parameters of each model into account when testing differences in data likelihood between nested models.

For experiments 1, 3 and 4 we found that a model containing the main effect of accuracy was sufficient to model the observed data (no significant difference in log-likelihood between full model and sparse model; Table 1). A model without enumeration accuracy as a predictor (over-cut model), however, suffered from significantly less data likelihood compared to the respective sparse model (Table 1) for those three experiments. Enumeration accuracy was a highly significant predictor of perceived longer oddball durations for both long duration—experiments (1 & 3) and short duration—experiment 4 (all *Wald-z* > 2.5, all *p* < .014). The main effect of accuracy was only marginally significant in short duration—experiment 2 (*Wald-z* = 1.9; *p* < .062) and dropped during model selection (Table 1).

**Table 1:** Mixed-effects Logit Models of time judgments for experiments 1–5. First column: Model formulas for the sparse, full and over-cut models in each experiment. Each model predicts the outcome variable “time judgment” (*TSE*, longer or shorter response on each trial) as a function of subject-specific intercepts and slopes across the physical oddball duration (*1+ T|S*) or set size (*1+ N|S*) for experiment 5. The fixed effects vary between experiments and the respective notation is given with physical oddball duration (*T*), enumeration accuracy (*Acc*), task order (*Order*), dot number (*No*). The inclusion of each predictor’s main effect in the model is denoted with a plus. The inclusion of interaction effects between predictors in the model is denoted with a colon. The inclusion of all main and interaction effects between predictors in the model is denoted with a cross (i.e.: *T x Order x T* denotes the inclusion of all three main effects and all interactions between the predictors in the model). Second to fifth column: degrees of freedom (*df*), Akaike (*AIC*), Bayesian (*BIC*) and *log-Likelihood* information criteria of each model. Sixth column-upper row: effect size (in *Wald-z* values) and *p*-values of all significant fixed effects in the sparse model. Lower rows: Likelihood-ratio tests (LRT) between sparse and full model and between over-cut and sparse model (*χ* ^*2*^-test statistic, degrees of freedom (*df*) of *χ* ^*2*^-test statistic and corresponding *p*-value).

Experiment 1	df	AIC	BIC	Log-L.	Fixed effects		
Sparse M TSE = (1+T|S) + T + Acc + Order: T	7	1979.0	2018.4	-982.5		Wald-z	p
					T	7.6	.001
					Acc	2.9	.004
					Order x T	2.7	.008
					other	<+/-1.4	n.s.
Full M TSE = (1+T|S) + T x Order x Acc	11	1977.8	2039.6	-977.9	LRT Sparse M / Full M		
					χ^2^	df	p
					9.2	4	.056
Over-cut M TSE = (1+T|S) + T + Order: T	6	1995.1	2028.9	-991.6	LRT Over-cut M / Sparse M		
					χ^2^	df	p
					18.1	1	.001
**Experiment 2**							
Sparse M TSE = (1+T|S) + T + Order: T	6	1658.5	1692.2	-823.3		Wald-z	p
					T	10.1	.001
					Order x T	3.1	.002
					other	<+/-1.3	n.s.
Full M TSE = (1+T|S) + T x Order x Acc	11	1663.5	1725.4	-820.8	LRT Sparse M / Full M		
					χ^2^	df	p
					5.0	5	.415
Over-cut M TSE = (1+T|S) + T	5	1666.4	1694.5	-828.2	LRT Over-cut M / Sparse M		
					χ^2^	df	p
					9.9	1	.002
**Experiment 3**							
Sparse M TSE = (1+T|S) + T + Acc	6	6022.1	6061.0	-3005.1		Wald-z	p
					T	4.8	.001
					Acc	4.3	.001
					other	<+/-1.1	n.s.
Full M TSE = (1+T|S) + T x No x Acc	11	6026.6	6097.8	-3002.3	LRT Sparse M / Full M		
					χ^2^	df	p
					5.5	5	.354
Over-cut M TSE = (1+T|S) + T	5	6038.7	6071.1	-3014.3	LRT Over-cut M / Sparse M		
					χ^2^	df	p
					18.6	1	.001
**Experiment 4**							
Sparse M TSE = (1+T|S) + T + Acc	6	3792.4	3828.7	-1890.2		Wald-z	p
					T	5.9	.001
					Acc	2.5	.014
					other	<+/-1.3	n.s.
Full M TSE = (1+T|S) + T x No x Acc	11	3793.5	3860.0	-1885.8	LRT Sparse M / Full M		
					χ^2^	df	p
					8.9	5	.112
Over-cut M TSE = (1+T|S) + T	5	3796.5	3826.7	-1893.2	LRT Over-cut M / Sparse M		
					χ^2^	df	p
					6.1	1	.014
**Experiment 5**							
Sparse M TSE = (1+No|S) + Acc	5	2621.0	2648.9	-1305.5		Wald-z	p
					Acc	3.1	.002
					other	<+/-0.8	n.s.
Full M TSE = (1+No|S) + No x Acc	7	2624.2	2663.2	-1305.1	LRT Sparse M / Full M		
					χ^2^	df	p
					0.8	2	.662
Over-cut M TSE = (1+No|S) + No	5	2629.7	2657.6	-1309.9	LRT Over-cut M / Sparse M		
					χ^2^	df	p
					6.1	1	.014

As expected, for all four experiments the best-fitting sparse model contained a term for physical oddball duration and for experiments 1 and 2 the response order of the tasks was a significant predictor of time judgments. In general, participants responses tracked the physical oddball duration more truthfully, likely due to increased attention towards time, when they judged the oddball duration first (see S1 Fig and S1 Results for the effect of response order on task performance). Oddball number was not a significant predictor of time judgments in experiments 3–5.

Similar results were found with a three-way within-subjects analysis of variance (ANOVA) on the proportions of longer responses for experiments 1 and 2 (factors oddball duration, enumeration accuracy and response order) and a two-way within-subjects ANOVA for experiment 3 and 4 (factors oddball duration and enumeration accuracy, Table 2). In sum, both logistic and linear models indicate that enumeration performance was an important predictor of perceived oddball duration in experiments 1,3 and 4.

**Table 2:** ANOVA models of time judgments for experiments 1–4. First column: Model formulas for the analysis of variance on the proportion of “longer” responses (TSE) for each experiment. The notation is the same as in Table 1. Second to fifth column: significant predictors in each ANOVA model with the degrees of freedom of the numerator (*df1*), denominator (*df2*), the *F*-test statistic and corresponding *p*-value.

		df1	df2	F	p
Experiment 1 TSE = T x Acc x Order	oddball time	3	45	240.2	.001
	accuracy	1	15	28.0	.001
	time x order	3	45	5.8	.002
	time x order x accuracy	3	45	4.7	.006
	other			< 3.2	n.s.
Experiment 2 TSE = T x Acc x Order	oddball time	3	45	218.7	.001
	time x order	3	45	6.7	.001
	other			< 2.1	n.s.
Experiment 3 TSE = T x Acc	oddball time	3	42	20.3	.001
	accuracy	1	14	5.0	.041
	other			< 2.5	n.s.
Experiment 4 TSE = T x Acc	oddball time	3	36	24.5	.001
	accuracy	1	12	13.3	.004
	other			< 1.4	n.s.

### 3.3. Time expansion & accuracy at the point of subjective equality: a critical test

**Fig 5:**
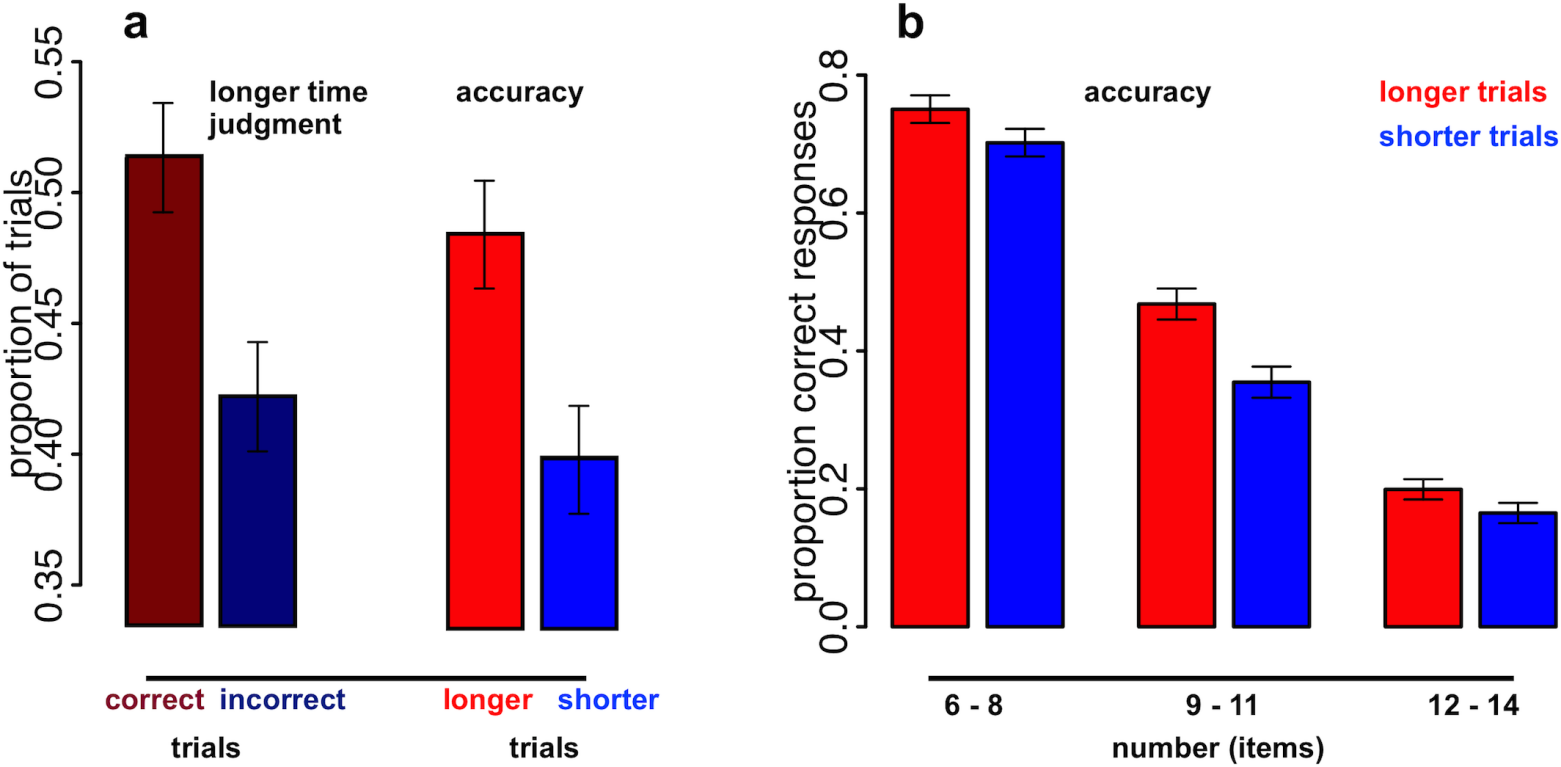
Time expansion & enumeration accuracy for oddballs around the PSE to standard duration. a: Oddballs were more often judged as perceptually “longer” than standard duration in correct compared to incorrect trials (dark red & blue bars). Conversely, oddballs were more often accurately counted in perceived longer compared to shorter trials (light red & blue bars). b: Oddballs perceived as perceptually “longer” than standard duration (light red bars) were more accurately counted compared to perceived “shorter” oddballs (light blue bars). This effect is particularly strong for intermediate set sizes (9–11 dots). (a & b) Error bars indicate one standard error of the mean (*SEM*) for within-subject designs (similar to the description in [26]). Individual proportions of correct responses of each subject have been centered on their average proportions of correct responses across conditions before calculating the *SE*.

The reported pattern of results suggests a functional link between time judgments and enumeration accuracy. When observers accurately counted the number of oddball dots, they perceive oddball durations as longer. In particular, enumeration accuracy was found to correlate highly with perceived temporal expansion (experiments 1 & 3). We hypothesized that oddball durations for which time judgments are at threshold, i.e. that yield an approximately equal number of “longer” and “shorter” responses corresponding to the PSE, should be particularly sensitive to this effect. As estimated from the previous experiment (see results 3.2.2), for durations around 1s the point of subjective equality (PSE) between standard (= 1050 ms) and oddballs across all trials was around 840 ms. With a different sample of observers, we now focused specifically on this oddball duration (840 ms) and measured its perceived duration (against standard duration (= 1050 ms)) and enumeration accuracy (within the range between 6–14 items) on each trial. On average, the oddball was perceived “longer” than the standard duration in 46.3% of all trials (*SD* = 9.2%; not different from 50%, *t*(12) = -1.5, *p* = n.s.), confirming the perceived equality of oddball and standard duration across all trials. Irrespective of its perceived duration (“longer/shorter”), the number of dots within each oddball display was accurately counted in 43.9% of all trials (*SD* = 9.7%). Critically, however, enumeration was significantly more accurate on trials in which temporal expansion occurred (“oddball > standard” responses), compared to trials with veridical time perception (“oddball < standard” responses; *F*(1,12) = 5.1, *p* < .05; Fig 5A and 5B). Likewise, the percentage of “longer” responses, signaling temporal expansion, was significantly higher in correct trials compared to incorrect trials (*t*(12) = 2.2, *p* < .05; Fig 5A) and the best-fitting logistic model predicting “longer” responses included an accuracy term (Table 1).

### 3.4. Perceived time as a function of number vs. accuracy

As described above, there was not a direct mapping between numerical and temporal magnitude judgments. Previous studies, however, reported interactions between perceived time and perceived number [34, 35] and we also found a small effect of perceived time for intermediate set sizes (see 3.1.3, Fig 2B and 3.3), which might have contributed at least partly to the observed findings. Therefore, we compared the modeling results in experiments 3–5, in which we presented a broad range of stratified set sizes, to formulas that model time judgments as a function of presented (N), reported (R) or the difference between reported and presented number (D). In all three experiments, the accuracy model (Acc) represented the empirical data best compared to the respective number models (N, R, D), as indexed by Akaike (AIC), Bayesian (BIC) and log-Likelihood information criteria (see S1, S2 and S3 Tables). We tested these results statistically with nested model comparison using likelihood ratio tests. For all three experiments, the accuracy model (alone without the respective number term) was not inferior to the parent model containing both accuracy and number main effects. The number model (alone without the accuracy term), however, suffered from significantly less data likelihood compared to the corresponding parent model for all three number predictors (presented (N), reported number (R) and number difference (D), see S1, S2 and S3 Tables). In sum, these findings suggest that oddball durations were consistently judged longer when enumeration was accurate and not when more/less items were presented or perceived.

## Discussion

As expected, participants showed a strong temporal oddball effect. The main novel finding was that participants showed better enumeration performance when time was perceived as expanded, while they performed worse when time was perceived as compressed. Thus, there was a direct relationship between accuracy and time, both in the case of objective time (better performance for longer oddball durations) and for subjective time (better performance when time was perceived as longer). Importantly, participants showed a specific effect of perceived duration: the largest effect was found beyond the subitizing range and when participants experienced strong time illusions.

In terms of potential mechanisms, the current results are consistent with multiple theories [1–3]. As mentioned above, a previous study measuring the effect of a click train on reaction times and memory performance argued that the train of clicks effectively “sped up” the rate of information processing [15]. According to models of temporal duration judgments based on an internal clock or pacemaker [11–13], the oddball might attract attention or increase arousal, leading both to faster processing of the oddball stimulus and longer perceived time. The close relationship between perceived duration and amount of information processed could reflect a parallel effect of arousal and/or attention on both an internal clock [13, 36] and sensory processing ([37, 38], for review see [39]). If perceived duration depends on the rate of such an internal clock then the increased arousal for an oddball could be expected to lengthen subjective passing of time. Likewise, accurately and precisely enumerating the items just beyond the subitizing range is likely to require focused attention. Attentional allocation of processing resources might therefore underlie both outcomes in temporal and visual tasks [40]. Future research is needed to tease apart the exact mechanisms underlying the oddball effect on both subjective time duration and enumeration performance.

An alternative explanation would be that rather than a parallel effect resulting from a common cause, the change in one task (perceived duration or perceived numerosity) directly affects performance in the other task. For example, the magnitude of time and number might map onto each other, or at least interact, as suggested by several studies ([34, 35, 41, 42]; but see also [43] and [44]). We did not find any evidence for a direct mapping between set size and perceived duration: more dots were not perceived as longer (Fig 2). However, there was a small, but complex, interaction between the number of items and temporal judgments in some experiments. Future studies are needed to better elucidate this relationship between temporal distortion and numerical cognition.

### Alternative explanations

Given that there is currently no agreement over how duration is judged, there are a number of alternative hypotheses about why the oddball effect might occur and how it would be related to enumeration performance. The first alternative explanation for this pattern of results that we can discard is that participants simply responded “long” when they felt like the task was easier since they inferred that they had more time to do the enumeration task (although see below for the exact opposite prediction). If so, then small numerosities should have been judged as longer, which was not the case, and they should have responded “longer” for correct trials across all oddball durations, which also did not occur. Instead, our effects were quite specific. Moreover, it is important to note that there was no feedback on whether participants were correct or not and average performance for intermediate numerosities (where we found the effect) ranged between 50%–70%, so clearly participants were likely not able to know whether they were correct or not. The temporal duration task was generally easier than the enumeration task. For the intermediate numerosities, the enumeration task was always hard, yet duration judgments generally tracked the actual duration of the oddball target (although with the added oddball effect).

The overall pattern of results found here cannot be explained by a more general relationship between effort and experienced duration [45]. Although it seems plausible that doing something difficult would seem to take more time than doing something easy, such a non-specific effect would have been expected to result in the opposite results to what we found: participants should have thought time was longer when there were more items in the display and when they got the answer wrong. Thus, neither of the simple explanations about task difficulty (harder means longer or harder means shorter) can account for the specific group of results found here.

In summary, the hypothesis that perceived duration and information processing are related, such that time feels expanded when input is processed effectively or temporal expansion allows for more accurate performance, is a parsimonious account for our data. It is consistent with the idea that the amount of information processing that occurs in a particular amount of objective time (the rate of information processing) is not constant [15], but can be greater or less as a function of factors like attention, arousal and the phase of ongoing neural oscillations [46]. When perceptual processing was more efficient, participants were likely to perceive time as expanded compared to when their performance in the enumeration task was worse.

### Conclusions

Overall, the results imply that subjective time distortions reflect real changes in sensory processing of the incoming stimulus. In other words, when time subjectively expands during unexpected events, it might allow for processing more information, or processing information more effectively, in the same objective temporal duration. Being able to vary the use of mental and physiological resources would be an efficient strategy to deal with the fact that life typically involves long periods of relative predictability and consistency interspersed with fewer moments in which we must quickly think and act. It would be wasteful to run our cognitive systems at 100% during the entire waking day. The ability to conserve resources when they are not needed but increase the efficiency of information-processing when needed would of course be evolutionarily advantageous in optimizing perception, thought and action during critical moments.

## Supporting Information

S1 Fig Time judgment as a function of response order in experiments 1 & 2. Proportion of longer responses as a function of oddball duration in experiments 1 (Figure a) and 2 (Figure b) when participants had to respond first the number judgment (blue) or the time judgment (red). Vertical, dashed lines indicate the standard duration (1050 ms in a; 70 ms in b). Red and blue dashed lines indicate one standard error of the mean (*SEM*).
(PDF)

S1 Results Time judgment as a function of response order in experiments 1 & 2. (DOCX)

S1 Table Mixed-effects Logit Models—Accuracy vs. Number for experiment 3. (DOCX)

S2 Table Mixed-effects Logit Models—Accuracy vs. Number for experiment 4. (DOCX)

S3 Table Mixed-effects Logit Models—Accuracy vs. Number for experiment 5. (DOCX)

## References

[1] Block RA, Zakay D (1997) Prospective and retrospective duration judgments: A meta-analytic review. Psychon B Rev 4: 184–197. 10.3758/BF03209393 21331825

[2] Zakay D, Block RA (1997) Temporal cognition. Curr Dir Psychol Sci 6: 12–16.

[3] Wittmann M (2009) The inner experience of time. Philos T R Soc B 364: 1955–1967. 10.1098/rstb.2009.0003 PMC2685813 19487197

[4] Arstila V (2012) Time slows down during accidents. Front Psychol 3: 196 10.3389/fpsyg.2012.00196 22754544 PMC3384265

[5] Campbell LA, Bryant RA (2007) How time flies: a study of novice skydivers. Behav Res Therapy 45: 1389–1392. 10.1016/j.brat.2006.05.011 16860291

[6] Stetson C, Fiesta MP, Eagleman DM (2007) Does time really slow down during a frightening event? PloS One 2: e1295 18074019 10.1371/journal.pone.0001295 PMC2110887

[7] Tse PU, Intriligator J, Rivest J, Cavanagh P (2004) Attention and the subjective expansion of time. Percept Psychophys 66: 1171–1189. 15751474 10.3758/bf03196844

[8] Ulrich R, Nitschke J, Rammsayer T (2006) Perceived duration of expected and unexpected stimuli. Psychol Res 70: 77–87. 15609031 10.1007/s00426-004-0195-4

[9] Van Wassenhove V, Buonomano DV, Shimojo S, Shams L (2008) Distortions of subjective time perception within and across senses. PloS One 3: e1437 10.1371/journal.pone.0001437 18197248 PMC2174530

[10] Seifried T, Ulrich R (2010) Does the asymmetry effect inflate the temporal expansion of odd stimuli?. Psychol Res 74: 90–98. 10.1007/s00426-008-0187-x 19034503

[11] Gibbon J (1977) Scalar expectancy theory and Weber’s law in animal timing. Psychol Rev 84: 279–325.

[12] Gibbon J, Church RM, Meck WH (1984) Timing and Time Perception. New York: The New York Academy of Sciences. 10.1111/j.1749-6632.1984.tb23417.x 6588812

[13] Treisman M (1963) Temporal discrimination and the indifference interval: Implications for a model of the" internal clock". Psychol Monogr–Gen A 77: 1. 10.1037/h0093864 5877542

[14] Buhusi CV, Meck WH (2005) What makes us tick? Functional and neural mechanisms of interval timing. Nature 6: 755–765 10.1038/nrn1764 16163383

[15] Jones LA, Allely CS, Wearden JH (2011) Click trains and the rate of information processing: does “speeding up” subjective time make other psychological processes run faster?. The Quarterly Journal of Experimental Psychology 64: 363–380. 10.1080/17470218.2010.502580 20737353

[16] Penton-Voak IS, Edwards H, Percival A, Wearden JH (1996) Speeding up an internal clock in humans? Effects of click trains on subjective duration. J Exp Psychol: Animal Behav Proc 22: 307–320. 10.1037//0097-7403.22.3.307 8691161

[17] Jevons WS (1871) The power of numerical discrimination. Nature 3: 363–372.

[18] Kaufman EL, Lord MW, Reese T, Volkmann J (1949) The discrimination of visual number. Am J Psychol 62: 496–525. 15392567

[19] Cowan N (2000) The magical number 4 in short-term memory: a reconsideration of mental storage capacity. Behav Brain Sci 24: 87–185. 10.1017/s0140525x01003922 11515286

[20] Ullman S (1984) Visual routines. Cognition 18: 97–159. 6543165 10.1016/0010-0277(84)90023-4

[21] Wutz A, Melcher D (2014) The temporal window of individuation limits visual capacity. Front Psychol 5. 10.3389/fpsyg.2014.00952 PMC4145468 25221534

[22] Wearden JH (2008) The perception of time: basic research and some potential links to the study of language. Language Learning 58: 149–171.

[23] Ivry RB, Spencer RMC (2004) The neural representation of time. Current Opinion in Neurobiology 14: 225–232 15082329 10.1016/j.conb.2004.03.013

[24] Brainard DH (1997) The psychophysics toolbox. Spatial Vision 10: 433–436. 9176952

[25] Pelli DG (1997) The VideoToolbox software for visual psycho-physics: Transforming numbers into movies. Spatial Vision 10: 437–442. 9176953

[26] Cousineau D (2005) Confidence intervals in within-subject designs: A simpler solution to Loftus and Masson’s method. Tutorials in Quantitative Methods for Psychology 1: 42–45.

[27] Wichmann FA, Hill NJ (2001) The psychometric function: I. Fitting, sampling, and goodness of fit. Percept Psychophys 63: 1293–1313. 11800458 10.3758/bf03194544

[28] Wichmann FA, Hill NJ (2001) The psychometric function: II. Bootstrap-based confidence intervals and sampling. Percept Psychophys 63: 1314–1329. 11800459 10.3758/bf03194545

[29] Jaeger TF (2008) Categorical data analysis: Away from ANOVAs (transformation or not) and towards logit mixed models. J Mem Lang 59: 434–446. 19884961 10.1016/j.jml.2007.11.007 PMC2613284

[30] Agresti A (2002) Categorical data analysis John Wiley & Sons.

[31] Breslow NE, Clayton DG (1993) Approximate inference in generalized linear mixed models. J Am Stat Assoc 88: 9–25.

[32] Bates D, Maechler M, Bolker B, Walker S, Bojesen Christensen RH, Singmann H, et al (2013) Package ‘lme4’: Linear mixed-effects models using S4 classes.

[33] Harrell JFE (2013) Package rms: Regression Modeling Strategies.

[34] Droit-Volet S, Clement A, Fayol M (2003) Time and number discrimination in a bisection task with a sequence of stimuli: A developmental approach. J Exp Child Psychol 84: 63–76. 12553918 10.1016/s0022-0965(02)00180-7

[35] Lambrechts A, Walsh V, van Wassenhove V (2013) Evidence accumulation in the magnitude system. Plos One: e82122 10.1371/journal.pone.0082122 24339998 PMC3855382

[36] Treisman M, Faulkner A, Naish PL, Brogan D (1990) The internal clock: Evidence for a temporal oscillator underlying time perception with some estimates of its characteristic frequency. Perception 19: 705–743. 2130371 10.1068/p190705

[37] Carrasco M, McElree B (2001) Covert attention accelerates the rate of visual information processing. P Natl Acad Sci USA 98: 5363–5367. 10.1073/pnas.081074098 PMC33215 11309485

[38] Maljkovic V, Martini P (2005) Short-term memory for scenes with affective content. J Vision 5: 215–299. 10.1167/5.3.6 PMC1307514 15929647

[39] Esyenck MW (1982) Attention and arousal, cognition and performance New York: Springer-Verlag.

[40] Thomas EA, Weaver WB (1975) Cognitive processing and time perception. Percept Psychophys 17: 363–367.

[41] Walsh V (2003) A theory of magnitude: common cortical metrics of time, space and quantity. Trends Cogn Sci 7: 483–488. 14585444 10.1016/j.tics.2003.09.002

[42] Dormal V, Seron X, Pesenti M (2005). Numerosity-duration interference: A stroop experiment. Acta Psychol 121: 109–124. 10.1016/j.actpsy.2005.06.003 16095549

[43] Gallistell RC, Gelman R (2000) Non-verbal numerical cognition: from reals to integers. Trends Cogn Sci 4: 59–65. 10652523 10.1016/s1364-6613(99)01424-2

[44] Agrillo C, Ranpura A, Butterworth B (2010) Time and numerosity estimation are independent: Behavioral evidence for two different systems using a conflict paradigm. Cogn Neurosci 1: 96–101. 10.1080/17588921003632537 24168275

[45] Marchetti G (2009) Studies on time: a proposal on how to get out of circularity. Cogn Process 10: 7–40. 10.1007/s10339-008-0215-1 18504631

[46] Wutz A, Weisz N, Braun C, Melcher D (2014) Temporal Windows in Visual Processing:“Prestimulus Brain State” and “Poststimulus Phase Reset” Segregate Visual Transients on Different Temporal Scales. J Neurosci 34: 1554–1565. 10.1523/JNEUROSCI.3187-13.2014 24453342 PMC3898303

