# Supplementary figures and images for: Expansion and Compression of Time Correlate with Information Processing in an Enumeration Task
Source: PLoS One. 2015 Aug 26;10(8):e0135794. doi: 10.1371/journal.pone.0135794 (PMC4550287; doi:10.1371/journal.pone.0135794)

proportion longer responses

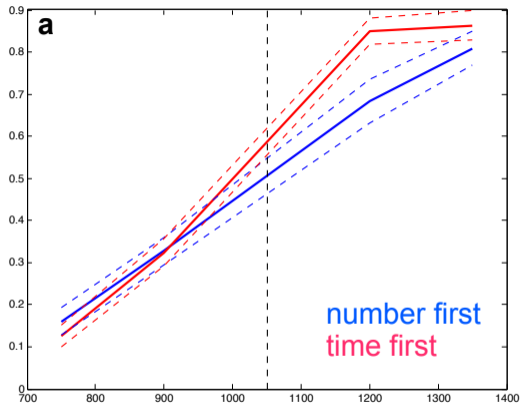

oddball duration (ms)

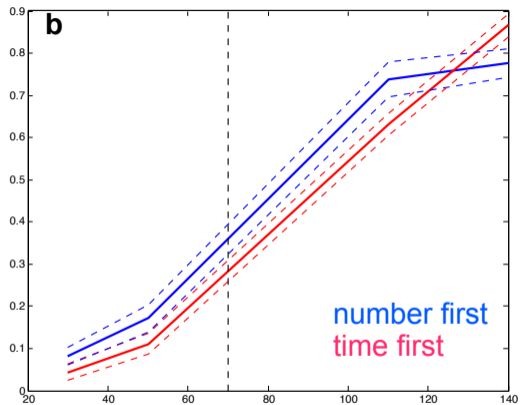

Supplement: S1 Fig — Proportion of longer responses as a function of oddball duration in experiments 1 (Figure a) and 2 (Figure b) when participants had to respond first the number judgment (blue) or the time judgment (red). Vertical, dashed lines indicate the standard duration (1050 ms in a; 70 ms in b). Red and blue dashed lines indicate one standard error of the mean (SEM). (PDF) [file pone.0135794.s001.pdf]
