# Supplementary material for: Expansion and Compression of Time Correlate with Information Processing in an Enumeration Task
Source: PLoS One. 2015 Aug 26;10(8):e0135794. doi: 10.1371/journal.pone.0135794 (PMC4550287; doi:10.1371/journal.pone.0135794)
Supplement: S1 Results — (DOCX) [file pone.0135794.s002.docx]

S1 results. Time judgment as a function of response order in experiments 1 & 2

In experiment 1 the two main factors “oddball duration” and “response order” interacted when predicting the time judgment (shorter or longer than standard duration) across participants. For durations below the standard duration there was no difference in the time judgments between response orders (all abs(*t*(15)<1.1, all *p* = n.s.). However, when the oddball duration was longer than the standard duration (1250 ms), participants reported this more often when they gave the time judgment first (*t*(15)=-3.5, *p* < .003). This finding is most likely due to increased attention on the oddball duration and hence more accurate performance when giving the time judgment first. Responding the number judgment first as in experiment 3, however, yields less “longer” responses when time expansion was expected and hence is the more conservative response order when estimating temporal expansion based on the proportion of longer responses.

The ANOVA (reported in table 2) also indicated a significant interaction between response order, oddball duration and accuracy. The logistic model (reported in table 1) did not show this effect, presumably because this triple interaction is based on a very limited number of trials. For both response orders the proportion of longer responses was higher in correct compared to incorrect trials across almost all oddball durations. This effect was stronger for longer oddballs (1250 and 1350 ms; both *t*(15)>3, both *p* < .008) when participants responded first in the number task but significant for both shorter and longer oddballs when participants responded first in the time task (750 and 1350 ms; both *t*(15)>2.2, both *p* < .05). Thus the ordinal order of the main effect of accuracy on the proportion of longer responses is preserved despite this triple interaction but alternating the response orders of the two tasks biased the strength of this effect across the different oddball durations.

In experiment 2 the two main factors “oddball duration” and “response order” also interacted when predicting the time judgment (shorter or longer than standard duration) across participants. When participants responded the time judgment first, they tended to respond more often that the oddball is shorter than the standard (for 30 ms and 110 ms oddballs, both *t*(15)>2.5, both *p* < .025) except for the longest oddball duration where we found the opposite effect (*t*(15)=-3.7, *p* < .0025). Similarly, this pattern of results is expected due to increased temporal attention when giving the time judgment first. Again when estimating time compression based on the proportion of longer responses, responding the number judgment first, as in experiment 4, yields less “shorter” responses and constitutes the more conservative response order.
