# Supplementary material for: Expansion and Compression of Time Correlate with Information Processing in an Enumeration Task
Source: PLoS One. 2015 Aug 26;10(8):e0135794. doi: 10.1371/journal.pone.0135794 (PMC4550287; doi:10.1371/journal.pone.0135794)
Supplement: S3 Table — (DOCX) [file pone.0135794.s005.docx]

| TSE = (1+N\|S) + term | df | AIC | BIC | log - Likelihood |
| --- | --- | --- | --- | --- |
| Acc | 5 | 2621.0 | 2648.9 | -1305.5 |
| No | 5 | 2629.7 | 2657.6 | -1309.9 |
| R | 5 | 2630.5 | 2658.4 | -1310.3 |
| D | 5 | 2631.2 | 2659.0 | -1310.6 |
|  |  |  |  |  |
| Likelihood ratio tests | | χ^2^ | df | p |
| Acc vs Acc + No |  | 0.2 | 1 | .626 |
| Acc vs Acc + R |  | 0.2 | 1 | .693 |
| Acc vs Acc + D |  | 0.004 | 1 | .950 |
|  |  |  |  |  |
| No vs Acc + No |  | 8.9 | 1 | .003 |
| R vs Acc + R |  | 9.7 | 1 | .002 |
| D vs Acc + D |  | 10.1 | 1 | .002 |

S3 Table. Mixed-effects Logit Models – Accuracy vs. Number for experiment 5

Upper table, first column: Model formulas for the accuracy (Acc), presented number (N), reported number (R) and number difference model (D). Each model predicts the outcome variable “time judgment” (*TSE*) as a function of subject-specific intercepts and slopes across set size (*1+ N|S*) and the main effect as specified in each model. Second to fifth column: degrees of freedom (*df*), Akaike (*AIC*), Bayesian (*BIC*) and *log-Likelihood* information criteria of each model.

Lower table, first column: Model formulas for nested model comparison using likelihood ratio tests (LRTs). Upper three rows denote LRTs between the accuracy model (without a number term) against the parent model (with both accuracy and number predictors) and lower three rows denote LRTs between each corresponding number model (without an accuracy term) against the parent model. Second to fourth column: *χ^2^*-test statistic, degrees of freedom (*df*) of *χ^2^*-test statistic and corresponding *p*-value. Accuracy models fit equally well compared to parent models but number models suffer from significantly less data likelihood.
